# Defining the Inflammatory Microenvironment in the Human Cochlea by Perilymph Analysis: Toward Liquid Biopsy of the Cochlea

**DOI:** 10.3389/fneur.2019.00665

**Published:** 2019-06-25

**Authors:** Athanasia Warnecke, Nils K. Prenzler, Heike Schmitt, Kerstin Daemen, Jana Keil, Martin Dursin, Thomas Lenarz, Christine S. Falk

**Affiliations:** ^1^Department of Otolaryngology, Hannover Medical School, Hanover, Germany; ^2^Cluster of Excellence of the German Research Foundation (DFG; “Deutsche Forschungsgemeinschaft”) “Hearing4all”, Oldenburg, Germany; ^3^Hannover Medical School, Institute of Transplant Immunology, Hanover, Germany

**Keywords:** hearing loss, neuroinflammation, cochlear implantation, cytokines, chemokines, endothelial factors, perilymph

## Abstract

The molecular pathomechanisms in the majority of patients suffering from acute or progressive sensorineural hearing loss cannot be determined yet. The size and the complex architecture of the cochlea make biopsy and in-depth histological analyses impossible without severe damage of the organ. Thus, histopathology correlated to inner disease is only possible after death. The establishment of a technique for perilymph sampling during cochlear implantation may enable a liquid biopsy and characterization of the cochlear microenvironment. Inflammatory processes may not only participate in disease onset and progression in the inner ear, but may also control performance of the implant. However, little is known about cytokines and chemokines in the human inner ear as predictive markers for cochlear implant performance. First attempts to use multiplex protein arrays for inflammatory markers were successful for the identification of cytokines, chemokines, and endothelial markers present in the human perilymph. Moreover, unsupervised cluster and principal component analyses were used to group patients by lead cytokines and to correlate certain proteins to clinical data. Endothelial and epithelial factors were detected at higher concentrations than typical pro-inflammatory cytokines such as TNF-a or IL-6. Significant differences in VEGF family members have been observed comparing patients with deafness to patients with residual hearing with significantly reduced VEGF-D levels in patients with deafness. In addition, there is a trend toward higher IGFBP-1 levels in these patients. Hence, endothelial and epithelial factors in combination with cytokines may present robust biomarker candidates and will be investigated in future studies in more detail. Thus, multiplex protein arrays are feasible in very small perilymph samples allowing a qualitative and quantitative analysis of inflammatory markers. More results are required to advance this method for elucidating the development and course of specific inner ear diseases or for perioperative characterization of cochlear implant patients.

## Introduction

The inner ear harbors the sensory organs responsible for balance and hearing. For many decades, the inner ear was assumed to be an immune-privileged organ because the blood-labyrinth barrier largely excludes major systemic components of the inflammatory response from the cochlear microenvironment (1). Despite this tight junction blood-labyrinth barrier and the absence of a lymphatic drainage, classical local, and systemic inflammatory mechanisms have been identified in the cochlea. The responsiveness of some types of rapidly progressing hearing loss to steroids and immunosuppressive treatment (2) was the first discovery to challenge the idea of the cochlea as an immune-privileged organ. It was acknowledged that antibody- as well as T cell-mediated responses are involved in the onset or progression of some types of hearing loss (3). In addition, myeloid cells have been identified in several compartments of the murine inner ear, including the stria vascularis (4), the spiral ligament (5), and the spiral ganglion (5). Acute damage to the murine inner ear caused by noise or ototoxic medication was shown to induce inflammation (6, 7) and to increase cochlear macrophages and neutrophils in the stria vascularis and the spiral ganglion (4, 8). Live imaging of adult mouse utricles revealed a phagocytic removal of cellular debris initiated by supporting cells following structural and cellular damage (9). An up regulation of immune-related genes in the murine cochlea has been also reported after noise exposure (10).

In general, three types of immune responses can be differentiated based on the involvement of various immune cells and soluble immune mediators (SIM): the innate immune system, which may be involved rather early in the onset of inflammatory responses and the adaptive specific immune system at later stages associated with disease progression. In the absence of infection, a third type of immune response coined sterile inflammation may be initiated after cell damage leading to an inflammatory response of the tissue. Cells of the innate immune system can sense microorganisms and viruses by pattern-recognition receptors (PRRs) enabling the detection of bacterial and fungal cell-wall components and viral nucleic acids termed as conserved pathogen-associated molecular patterns (PAMPs) (11, 12). This leads to further recruitment of innate effector cells, i.e., neutrophils, monocytes/macrophages, dendritic cells, and natural killer cells, to the inflammatory site and this process is mainly mediated by inflammatory chemokines (13). In contrast, the adaptive T cell-mediated immune response is initiated after sensing of PAMPs by PRRs and antigen presentation, i.e., peptides in the context of major histocompatibility complex (MHC), particularly by dendritic cells (12). Migration of antigen presenting cells (APC) into the draining lymph node is important for activation of antigen (pathogen) -specific effector and helper T cells and antibody-producing B cells, respectively (13). However, in the absence of infection, it is currently unknown whether and how immune activation may be involved in the development of deafness.

One potential mechanism could be sensing of danger associated molecular pattern (DAMPs), which are mainly DNA, nuclear, and heat shock proteins released from damaged tissue cells by innate immune cells. Hence, sterile cell death or tissue injury can induce inflammation related to innate immune responses to pathogens (11). Usually, DAMPs are removed intracellularly under physiological conditions and remain undetected by the immune system. However, pathological conditions such as injury or oxidative stress lead to the release of DAMPs that might be extracellular in the first line such as fragments of the extracellular matrix that occur after severe damage or by proteases activated to promote tissue repair and remodeling (11).

In general, cochlear implantation causes trauma by opening of the cochlea and insertion of an electrode array. As a consequence, immunological and tissue repair mechanisms may occur and result in severe damage of the residual cochlear structural and ultrastructural components (14–17). Clinically, this results in loss of residual hearing after cochlear implantation (18–20). After damage, tumor necrosis factor alpha (TNF-α) was shown to be expressed and released by cells of the stria vascularis and the spiral ligament (8) and stimulate a cascade of cytokine and chemokine expression leading to recruitment of immune cells and further damage. Actually, anti-inflammatory therapeutics like glucocorticoids are administered alongside implantation to reduce trauma and rescue residual organ function, however, with minimal long-term efficacy (21). Suppression of inflammatory pathways may trigger compensatory responses by activation of alternative pathways (22) since inflammation is a necessary reaction protecting the organ from excessive damage (22). Also, there is increasing evidence that excessive activation of the glucocorticoid pathway can be neurotoxic (23–26). Alternative therapeutic interventions should, therefore, aim at stabilizing the local cochlear environment by balancing endogenous pro- and anti-inflammatory reactions, thus, allowing full resolution of inflammation and recovery of organ homeostasis that results in preservation of residual organ structures and improved speech perception (1).

Autologous bone marrow-derived mononuclear cells can release immunomodulative factors and have a highly protective effect on cochlear cells as shown in previous work (27). In order to advance such therapeutic approaches to daily clinical routine, information about the inflammatory microenvironment of the individual cochlea that is implanted would be helpful. Since tissue sampling from the inner ear is not possible without excessive damage and loss of the organ, we have developed a liquid biopsy by collecting human perilymphatic fluid from the inner ear (28). This can be easily performed since the round window membrane is the natural sealing of the cochlear perilymphatic space. Since the round window will be opened anyway to allow insertion of the electrode array during cochlear implantation, it is not critical to puncture the round window membrane just prior to the opening by a microcapillary allowing the atraumatic collection of the inner ear fluid by capillary forces.

In the present study, we used this approach to analyse perilymph samples with an array of inflammatory markers resembling SIM involved in innate and the adaptive immune responses or sterile tissue inflammation as well as epithelial and endothelial regulators and to correlate these with residual cochlear function prior to cochlear implantation.

## Materials and Methods

The present study has been approved by the institutional ethical committee (approval no. 1883–2013). Perilymph was collected after written informed consent from the patients (in case of pediatric patients from their parents) receiving cochlear implantation between 09/17 and 05/18. A total of 43 patients (one being implanted bilaterally in two surgical sessions 3 weeks apart) resulting in a total of 44 perilymph samples were included. The collected sample volumes ranged from 1 to 4 μl. Demographic data of the patients are summarized in Table 1.

**Table 1:** Overview of demographic data of CI patients.

	**All patients (*n* = 44)**	**Pts. with surditas (SDT) *n* = 37**	**Residual hearing (<80 db) *n* = 7**	***P*-value**
Age (mean; range)	45.2 (0.7–87.1)	43.12 ± 4.8	56.0 ± 6.4	0.27 (n.s.)
Sex (m/f) percentages	26/18 (59/41%)	15/12 (56/44%)	5/2 (71/29%)	

### Perilymph Sampling

Human perilymph was collected with a customized glass microcapillary during cochlear implantation. For perilymph collection, the round window membrane was exposed by removal of its bony overhang and the intact membrane was punctured with the ultrathin and sharp tip of the glass capillary directly before the insertion of the electrode array. The collection was performed without any suctioning just by capillary forces. This resulted in different volumes of perilymph. The samples were left in the capillary and immediately transferred into a petri dish placed on ice. After transfer to the laboratory, the capillaries were connected to a pipette and the fluid was pipetted into small vials for freezing at −80°C until evaluation with a multiplex protein array.

### Classification of Patients by Audiogram Data

All patients assigned to cochlear implantation were evaluated audiologically with a defined battery of tests according to our and international standards. Pure tone audiometry (PTA) was performed prior to surgery only in adults and kids at school age. Additionally, otoacoustic emissions, auditory brainstem responses, and electrocochleography were performed in each patient pre-operatively.

The pre-operative audiograms were used to classify patients into two groups: Patients with no residual hearing with averages in PTA of 80 dB or higher at three contiguous frequencies (250, 500, and 750 Hz). Patients with PTA threshold of <80 dB were considered as having residual hearing. In children, the responses in ABR were used for the classification and all small children included had no ABR responses. The etiology of hearing loss was unknown in the majority of the patients. Six patients had congenital severe hearing loss, eight patients congenital onset of hearing loss with progression over time and late onset hearing loss with progression was observed in 13 cases. Menière's disease (*n* = 2), meningitis (*n* = 2), vestibular schwannoma (*n* = 1), and otosclerosis were among the known etiologies. One patient suffered from congenital hearing loss and glucogen storage disease type 1, one from progressive hearing loss and myasthenia gravis. Repeated attacks of sudden sensorineural hearing loss were reported by six patients and two patients suffered from single sided deafness.

### Quantification of Cytokine, Chemokine, and Tissue Factors Using Multiplex Protein Arrays

Concentrations of SIM and epithelial and endothelial factors were determined using Luminex-based multiplex arrays, i.e., human 27-Plex (M500KCAF0Y, BioRad, Hercules California, USA), and Cancer Panel 2 (171AC600M, BioRad) in a miniaturized variant of the manufacturer's instructions. As little as 1–2 μl of perilymph fluid were diluted with sample diluent (1:20) and incubated with multiplex beads for 45 min followed by two washings steps, cocktail of biotinylated secondary mAbs for 30 min and after final washing steps, streptavidin-PE was added. Greater than fifty beads per sample per analyte were detected using the BioPlex Manager 6.2 Software and concentrations were calculated according to individual standard curves for each analyte ranging from ~20 ng/ml to the detection limit of ~2 pg/ml.

### Unsupervised Cluster and Principal Component Analyses

The complete dataset of 43 analytes from 44 perilymph samples was analyzed using Qlucore Omics Explorer (Version 3.3, Lund, Sweden). Data were log2 transformed, scaled to mean zero, variable one, and threshold of 0.01. Discriminating variables were determined using linear models and multigroup ANOVA comparisons.

### Descriptive Statistical Analyses

D'Agostino-Pearson omnibus normality test was used to assess data distribution. Statistical analyses were performed as indicated in figure legends with *p* < 0.05 considered significant. All statistical analyses were calculated with GraphPad Prism (Version 6.07, La Jolla, USA).

## Results

**Figure 1:**
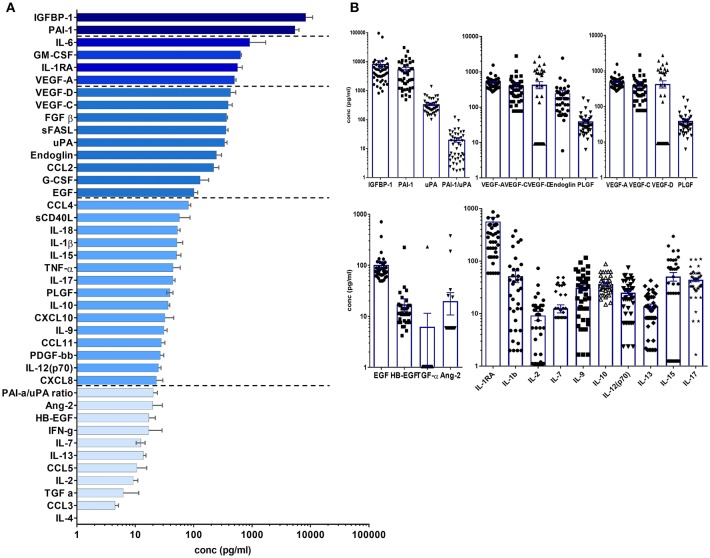
Cytokine/chemokine and tissue factor composition of human perilymph fluid. **(A)** Human perilymph fluid comprises high concentrations of epithelial and endothelial proteins like IGFPB1, VEGF-A,-C,-D, some innate cytokines like IL-6, followed by chemokines and typical cytokines of the adaptive immune system like IFN-g, I-17, etc. Concentrations were determined from *n* = 44 perilymph samples, median concentrations ± SEM are displayed in a waterfall plot. **(B)** In order to show that most proteins were detected in all patient, the concentrations of individual proteins are shown for each sample using log scale graphs.

Tissue factors and SIM were detectable in all perilymph samples of the 44 patients demonstrating the feasibility of the multiplex technology for small volumes below 5 μl perilymph fluid. A list of all proteins included in the human 27-plex and Cancer Panel 2 arrays is given in Table 2. Of note, a homogeneous concentration range was observed in all samples with the insulin-like growth factor binding protein 1 (IGFBP1) and the plasminogen activator inhibitor 1 (PAI-1) at very high concentrations (>1,000 pg/ml) followed by four proteins higher than 500 pg/ml, i.e., the cytokine IL-6, the granulocyte-macrophage colony stimulating factor (GM-CSF), the IL-1 receptor antagonist (IL-1RA), and vascular endothelial growth factor A (VEGF-A) (Figures 1A,B). Together with high concentrations of the urokinase plasminogen activator (uPA), the ratio of PAI-1/uPA (mean ratio 20.3) showed a remarkably homogenous distribution (Figure 1B). Additional endothelial and epithelial factors were detected at high concentrations between 100 and 500 pg/ml of VEGF-D, -C, endoglin, epidermal growth factor (EGF), fibroblast-growth factor beta (FGF-β), and IL-18, a caspase-1 dependent indicator of cell damage. This pattern further supported a tissue-related microenvironment within perilymph fluid accompanied by several immune activation markers like the soluble Fas (CD95) ligand (sFasL), the chemokine CCL2 (MCP-1), responsible for recruitment of myeloid cells, and the granulocyte colony stimulating factor G-SCF in the same concentration range. Of note, the classical cytokine regulators of T cell function like IL-9, IL-10, IL-12p70, IL-15, IL-17, TNF-α, and the soluble CD40 ligand (sCD40L) were detectable at concentrations below 100 pg/ml along with typical inflammatory chemokines like CXCL8, CXCL10, and CCL4 indicating a low but detectable degree of both innate and adaptive immune activation. Within this intermediate group, the pro-inflammatory innate cytokine IL-1β was detected at remarkable concentrations (mean 51.5 pg/ml), which was balanced by a 10-fold excess of IL-1RA (mean concentration 566.3 pg/ml). The low concentration group (<100 pg/ml) comprised T cell cytokines like IL-2, IL-4, IL-7, IL-13, IFN-γ, chemokines (CCL3, CCL5) as well as endothelial factors (Ang-2, HB-EGF, TGF-a) arguing rather for a tightly regulated specific microenvironment composed by tissue factors and selected SIM in cochlear perilymph fluid than for an uncontrolled pro-inflammatory milieu. While most proteins showed a homogeneous, though not always gaussean distribution, a bimodal distribution could be observed for others like IGFBP-1, VEGF-D, IL-1β, and IL-6.

**Table 2:** A list of all proteins included in the human 27-plex and cancer panel 2 arrays.

**Human 27-plex**	**Cancer panel 2**
FGF basic	Angiopoietin-2
Eotaxin	sCD40L
G-CSF	EGF
GM-CSF	Endoglin
IFN-γ	sFASL
IL-1β	HB-EGF
IL-1ra	IGFBP-1
L-2	IL-6
IL-4-IL10	IL-8
IL-12 (p70)	IL-18
IL-13	PAI-1
IL-15	PLGF
IL-17A	TGF-α
IP-10	TNF-α
MCP-1 (MCAF)	uPA
MIP-1α	VEGF-A
MIP-1β	VEGF-C
PDGF-BB	VEGF-D
RANTES	
TNF-α	
VEGF	

In order to exclude an impact of age on the composition of the cochlear microenvironment, a correlation matrix was calculated for all proteins vs. age with only minor negative correlations for sFasL (Spearman *r* −0.456), VEGF-C (Spearman *r* −0.3105), and FGF-b (Spearman *r* −0.34), and G-CSF (Spearman *r* −0.31, Supplementary Table 1). In addition, unsupervised hierarchical cluster and principal component analyses (PCA) revealed no correlation between age or gender and individual or particular groups of SIM or tissue factors within perilymph fluid (data not shown).

**Figure 2:**
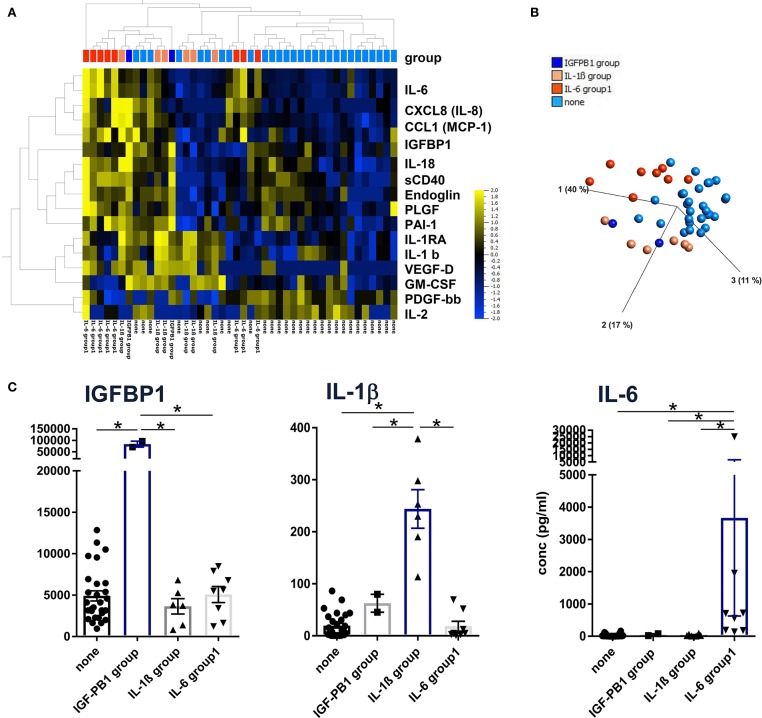
Definition of patient subgroups according to hierarchical clustering, principal component analyses. **(A)** Concentrations of 43 proteins from 44 perilymph samples were log-normalized with a threshold of 0.01 and filtered to *p* < 0.05 with *q* = 0.2 and visualized using hierarchical cluster and **(B)** PCA algorithms (Qlucore Omics 3.6 Software). Patient subgroups were defined by significantly high IGFBP1, IL-1b, and IL-6 concentrations, respectively, indicated by the color code shown in **(B)**. **(C)** Descriptive statistics were applied to the four patient groups using raw protein concentrations and Kruskal-Wallis tests with *p* < 0.05 defined as significant and marked with an asterisk (*).

Unsupervised hierarchical clustering and PCA methods with *p* < 0.05 and *q* < 0.1 ANOVA settings were applied to group patients according to the lead cytokines IL-1β and IL-6 and the epithelial protein IGFBP-1, respectively (Figures 2A,B). With this approach, patients could be assigned to one of the following groups defined by significantly higher concentrations of IGFBP1, IL-1β, IL-6, or none of these (Figure 2C). Although these three proteins were clustered in the heat map shown in Figure 2A, they were not coregulated in the patients since they displayed elevated levels only in the IGFBP1, IL-1b, or IL-6 group, respectively. Therefore, the different patterns seem to be characteristic for patient subgroups, which indicates diverse pathways of inflammation and tissue repair/regeneration, finally leading to complete or progressive sensorineural hearing loss.

**Figure 3:**
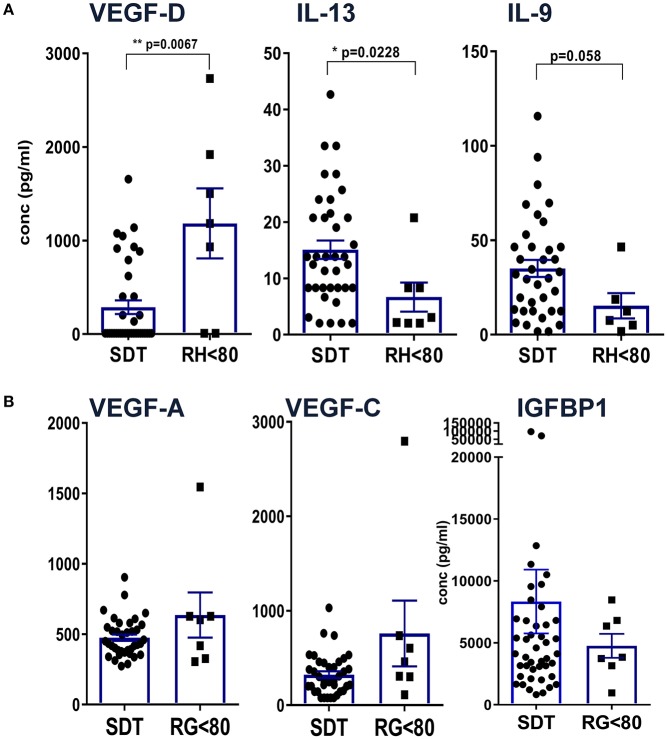
Definition of differences between patients with complete hearing loss vs. residual hearing capacity. Patients were grouped according to their acoustical performance at the time of CI with a hearing cut-off of <80 for patients with residual hearing (RH) vs. complete deafness (surditas SDT). Mann-Whitney-*u*-tests were performed for **(A)** VEGF-D, IL-13, and IL-9 and **(B)** VEGF-A, -C, and IGFBP1, respectively, with differences *p* < 0.05 defined as significant and marked with (*) and *p* < 0.001 with (**).

In order to uncover patterns related to residual hearing, CI patients were grouped into 37 individuals with no residual hearing as defined above as surditas (SDT) and seven patients with some residual hearing prior to implantation (RH, Figure 3). Among all tested proteins, significant differences were only detected for VEGF-D, IL-13, and IL-9 with lower VEGF-D levels in patients with no residual hearing compared to the group of patients with residual hearing (^**^p < 0.01; Figure 3A). In contrast, IL-13 and IL-9 concentrations were higher in patients with complete deafness (surditas, SDT) compared to patients with residual hearing (^*^p < 0.05; Figure 3A) although statistical significance was missed for IL-9 (*p* = 0.058). In contrast to VEGF-D, the other two ligands of VEGFR1/2, i.e., VEGF-A and -C did not differ between patients with complete or partial deafness (Figure 3B). Despite the broad concentration range of IGFBP1, a tendency was observed toward an increased concentration in patients with complete deafness, which was accompanied by a higher, though non-significantly, PAI-1/uPa ratio due to higher PAI-1 levels (data not shown). These patterns further support the idea of different pathways associated with progression of hearing loss and, hence, the possibility to identify biomarker candidates with relevance also for future treatment strategies in the course of implantation.

## Discussion

The approach of a multiplex-based protein analysis of human inner ear fluid was shown to be feasible and depicts a progress in hearing research where generally animal models were used. Here, we present an analytical method for the simultaneous detection and quantification of currently 43, but in the future up to 100 cytokines, chemokines, epithelial, and endothelial factors in tiny volumes of human perilymph. Surprisingly, the microenvironment in perilymph fluid was composed primarily by proteins regulating endothelial and epithelial functions followed by chemokines and cytokines (Figure 1A). With this strategy, we can characterize patterns of different immune responses allowing the definition of the cochlear inflammatory state in patients in which we can access the inner ear at the time of cochlear implantation. Some of the surgical approaches for the treatment of disorders affecting the inner ear enable collection of human perilymph and several centres use this technique for proteomics, metabolomics and microRNA detection (28–32). However, none of these previously reported techniques would allow the direct and simultaneous quantification of inflammatory and tissue markers.

Among the factors with the highest concentration, we identified regulators of tissue repair and angiogenesis along with inflammatory factors. The highly prevalent protein IGFBP1 regulates the action of insulin-like growth factor (IGF-1) by either enhancing or inhibiting its activity (33). IGF-1 plays a key role in embryonic and post-natal development of the cochlea (34) and has been identified as strong protective agent for hair cells (35). Indeed, local application of recombinant human IGF-1 was shown to be effective for the treatment and prevention of noise-induced hearing loss in guinea pigs (36) and in rats (37). Therefore, the broad range of IGFBP1 in CI patients indicates a tight regulation of the IGF receptor system. In addition, a synergy of the growth factors IGF-1 and EPO for acute neuroprotection by activation of the PI3-K-Akt pathway in cell culture experiments (33) has been described.

Plasminogen activator inhibitor-1 (PAI-1) is a pro-coagulant that is released by platelets and the endothelium in response to inflammation, damage, or ischemia and can be used as early marker for endothelial injury based on its inhibitory function of urokinase-like plasminogen activator (uPA) (38). Elevations of PAI-1 in bronchiolar lavage fluid is associated with experimentally-induced pulmonary fibrosis after thoracic radiation exposure (39) and in patients with lethal acute respiratory distress syndrome (40). Thus, the presence of PAI-1, uPA, and the high PAI-1/uPA ratio (>10) in perilymph may be an indicator for cochlear distress and damage. In our prior study, PAI-1 was detected by mass spectrometry only in one perilymph sample of a patient with hepatitis C infection and otosclerosis (28) underlying the hypothesis for an inflammation indicator in perilymph. Tissue plasminogen activator (tPA) and uPA cleave plasminogen to the serine protease plasmin (41). Plasmin is the most significant protease that cleaves pro-neurotrophins to the active neurotrophins, thus providing trophic support to auditory neurons and stabilization of synapses (41). PAI-1 inhibits tPA and uPA and whether the increased presence of PAI-1 in perilymph might be an indicator of cochlear health needs further evaluation.

Interleukin1-receptor antagonist (IL-1RA) is a potent factor to reverse effects mediated by the pro-inflammatory cytokines interleukin 1α and β in many diseases. For example, in an animal model of tendinopathy, IL1-RA can reduce pathological changes (42). Increased levels of IL1-RA can be used as a predictor for cardiovascular mortality in patients with acute coronary syndrome or stable angina (43). Recombinant IL-1RA (Anakinra) is a useful treatment for several autoimmune diseases. However, the role of IL1-RA in the inner ear is unclear to date and in this specialized compartment it could act as anti-fibrotic agent in CI and the risk of consecutive fibrosis needs thorough investigation.

Interleukin 6 initiates classical innate inflammatory pathways and, furthermore, inhibits neuronal proliferation and differentiation, thus decreasing neurogenesis in the adult brain (44). In the course of infections, IL-6 is necessary for B cell development into plasma cells and their antibody production. Of note, IL-6 expression from inner ear fibrocytes can be triggered by TNFα (45, 46). In a mouse model, elevated IL-6 levels exacerbated CMV-related cytotoxic and inflammatory injury of the inner ear (47). Therefore, IL-6 levels in human perilymph fluid may be involved in causing hearing deficits associated with infections or other inflammatory cascades.

Adhesion molecules and endothelial factors are also involved in inner ear repair and regeneration. Especially VEGF family members induce vascular neogenesis and increases the permeability of the blood-brain-barrier (48). In our study, comparing patients with complete deafness to patients with residual hearing, there was no difference in the concentrations of VEGF-A and -C but VEGF-D was reduced in patients with deafness. VEGF-A was the first discovered form of the vascular endothelial growth factors and is a potent regulator of angiogenesis. It is secreted by a variety of cells upon oxygen deprivation (49) causing cell proliferation, apoptosis inhibition, increased vascular permeability, and recruitment of inflammatory cells (50–52). By contrast, VEGF-C and VEGF-D have a central role in lymphangiogenesis with little function in vasculogenesis via binding to VEFR2 and 3 (53). Interestingly, the expression of VEGF is increased after damage to the inner ear (54, 55). Reduced levels of VEGF-D in patients with complete deafness compared to patients with some residual hearing may be an indicator for on-going damage and reduced repair in the inner ear. It is also possible that the specific isoform VEGF-D may have protective effects on the inner ear although it has been shown that VEGF without a distinction of its isoform has no protective effects on auditory hair cells (56). At present, we cannot define whether elevated VEGF-D levels may result from a compensatory mechanism that improves the capacity of the lymphatic vessels to remove fluid from the extravascular space (57).

Unexpectedly, higher IGFBP1 levels were measured in patients with complete loss of auditory function compared to patients with residual hearing. IGFBP1 represents an important regulator of insulin-like growth factors at the IGF receptor (58). Based on its general function for cell survival, the IGF pathway has been proposed as potential therapeutic target for the inner ear (59). Our previous finding of miRNAs involved in IGF signaling further supports the relevance of this pathway (32) along with this novel identification of another IGF regulator at the protein level.

The observation that classical Th1/Th2/Th17 cytokines were only detected at low concentrations compared to tissue factors and chemokines argues for a rather minor role of T cells for hearing loss at this late stage of cochlear implantation. Nevertheless, significantly higher concentrations of IL-13 and IL-9 were detected in perilymph of patients with complete loss compared to residual hearing, which would at least argue for an impact of Th2 and Th9 cytokines. Both cytokines can be induced by various stimuli including toxic substances via the aryl hydrocarbon receptor (AhR) that has recently been shown to act as transcription factor during development of the inner ear (60).

Taken together, our combined analysis of inflammatory and tissue regulatory proteins provides a more refined insight into the microenvironment of the inner ear. Several dysregulated pathways may lead to progressive sensorineural hearing loss and one possible scenario could be a combination of a toxic environment, sensed via AhR, further promoted under pro-inflammatory conditions and manifested by insufficient repair mechanisms that finally lead to complete deafness.

The following limitations of the presented study need to be taken into account. Contaminations with blood and cerebrospinal fluid (CSF) seem to be very unlike as discussed in our previous work (28). Surely, in the rare cases of a patent cochlear aqueduct, this may be different. Usually, such patients have a gusher and an increased yield of perilymph fluid during collection indicating the possibility of contamination with CSF. Among the patients included in this study, none had a gusher. Due to the small size of samples, the study is underpowered to analyse the association between cytokine networks and the degree of damage of the cochlea. Thus, this exploratory approach needs validation in large independent cohorts. In addition, only the baseline chemokine, cytokines, and growth factor profile can be assessed with the current perilymph sampling technology, not allowing for serial investigations. The study is also underpowered for any subgroup analyses due to the small size. Future studies will therefore, focus on the identification of cochlear inflammatory marker that are disease specific, represent the degree of the damage to the organ or may be even predictive for the performance that can be expected with a cochlear implant.

## Conclusion

Multiplex protein analyses are feasible in very small samples (~1 μl or less) of human perilymph fluid and is able to identify marker proteins of sterile inflammation as well as of the innate and adaptive immune system. Thus, this method may be advanced to a key perioperative characterization procedure for the inflammatory state of the cochlea in patients in need of cochlear implant. Knowledge about the state of the cochlea prior to implantation may offer an excellent tool for the deployment of novel adjuvant pharmacotherapies and may pave the avenue toward a new era of personalized medicine.

## Data Availability

This manuscript contains previously unpublished data. The name of the repository and accession number are not available.

## Ethics Statement

The present study has been approved by the institutional ethical committee (approval no. 1883–2013). Perilymph was collected after written informed consent from the patients (in case of pediatric patients from their parents) receiving cochlear implantation between 09/17 and 05/18.

## Author Contributions

AW and CF conception and design of the work, acquisition, analysis, interpretation of data for the work, drafting the work, and approval for publication of the content. NP and HS interpretation of data for the work, revising the manuscript critically for important intellectual content, and approval for publication of the content. KD and JK acquisition and analysis of data for the work and approval for publication of the content. MD and TL conception of the work, interpretation of data for the work, revising the manuscript critically for important intellectual content, and approval for publication of the content.

### Conflict of Interest Statement

The authors declare that the research was conducted in the absence of any commercial or financial relationships that could be construed as a potential conflict of interest.
