# Supplementary material for: Defining the Inflammatory Microenvironment in the Human Cochlea by Perilymph Analysis: Toward Liquid Biopsy of the Cochlea
Source: Front Neurol. 2019 Jun 25;10:665. doi: 10.3389/fneur.2019.00665 (PMC6603180; doi:10.3389/fneur.2019.00665)
Supplement: Supplementary file 1 [file Table_1.DOCX]

Supplementary Material

# Supplementary Figures and Tables

Supporting Table 1: correlation matrix of SIM and tissue factors to patient age

| age versus | Spearman r | 95% confidence interval | P (two-tailed) | P value summary | Exact or approximate P value? | Significant? (alpha = 0.05) |
| --- | --- | --- | --- | --- | --- | --- |
| Ang-2 | 0.1171 | -0,1949 to 0,4077 | 0,4489 | ns | approximate | No |
| sCD40L | 0.04423 | -0,2681 to 0,3481 | 0,7782 | ns | approximate | No |
| EGF | -0.1074 | -0,3995 to 0,2044 | 0,4876 | ns | approximate | No |
| Endoglin | -0.2127 | -0,4863 to 0,0988 | 0,1656 | ns | approximate | No |
| sFASL | **-0.456** | **-0,6682 to -0,1753** | **0,0019** | ****** | approximate | **Yes** |
| HB-EGF | -0.07196 | -0,369 to 0,2384 | 0,6425 | ns | approximate | No |
| IGFBP-1 | -0.1648 | -0,4474 to 0,1478 | 0,2851 | ns | approximate | No |
| IL-18 | -0.06323 | -0,3614 to 0,2466 | 0,6835 | ns | approximate | No |
| PAI-1 | -0.1728 | -0,454 to 0,1397 | 0,2619 | ns | approximate | No |
| PLGF | 0.05997 | -0,2497 to 0,3585 | 0,699 | ns | approximate | No |
| TGF a | 0.2102 | -0,1014 to 0,4842 | 0,1709 | ns | approximate | No |
| uPA | 0.1337 | -0,1787 to 0,4216 | 0,387 | ns | approximate | No |
| VEGF-A | -0.136 | -0,4235 to 0,1764 | 0,3788 | ns | approximate | No |
| VEGF-C | **-0.3105** | **-0,5623 to -0,005972** | **0,0402** | ***** | **approximate** | **Yes** |
| VEGF-D | -0.2454 | -0,5122 to 0,06455 | 0,1084 | ns | approximate | No |
| PAI-a/uPA ratio | -0.2274 | -0,498 to 0,08347 | 0,1376 | ns | approximate | No |
| IL-1β | -0.1035 | -0,3961 to 0,2082 | 0,5038 | ns | approximate | No |
| IL-1RA | -0.2643 | -0,5269 to 0,04442 | 0,083 | ns | approximate | No |
| IL-2 | 0.09335 | -0,218 to 0,3874 | 0,5467 | ns | approximate | No |
| IL-4 | 0.1076 | -0,2042 to 0,3996 | 0,487 | ns | approximate | No |
| IL-5 |  |  |  |  | approximate |  |
| IL-6 | -0.01198 | -0,3159 to 0,2942 | 0,9385 | ns | approximate | No |
| IL-7 | -0.2489 | -0,5149 to 0,0608 | 0,1032 | ns | approximate | No |
| CXCL8 | -0.2011 | -0,48 to 0,1147 | 0,196 | ns | approximate | No |
| IL-9 | 0.09231 | -0,219 to 0,3865 | 0,5512 | ns | approximate | No |
| IL-10 | -0.2625 | -0,5255 to 0,04634 | 0,0852 | ns | approximate | No |
| IL-12(p70) | -0.07932 | -0,3753 to 0,2314 | 0,6088 | ns | approximate | No |
| IL-13 | -0.2688 | -0,5304 to 0,03958 | 0,0777 | ns | approximate | No |
| IL-15 | -0.2045 | -0,4797 to 0,1073 | 0,183 | ns | approximate | No |
| IL-17 | -0.1416 | -0,4282 to 0,1709 | 0,3593 | ns | approximate | No |
| Eotaxin | -0.01131 | -0,3153 to 0,2948 | 0,9419 | ns | approximate | No |
| FGF β | **-0.34** | **-0,5845 to -0,03894** | **0,0239** | ***** | approximate | **Yes** |
| G-CSF | -0.1404 | -0,4272 to 0,172 | 0,3632 | ns | approximate | No |
| GM-CSF | **-0.3126** | **-0,5639 to -0,008233** | **0,0389** | ***** | approximate | **Yes** |
| IFN-γ | 0.2102 | -0,1014 to 0,4842 | 0,1709 | ns | approximate | No |
| CXCL10 | 0.1641 | -0,1485 to 0,4468 | 0,2873 | ns | approximate | No |
| CCL2 | -0.0636 | -0,3617 to 0,2463 | 0,6817 | ns | approximate | No |
| CCL3 | -0.1676 | -0,4497 to 0,145 | 0,2769 | ns | approximate | No |
| PDGF-bb | 0.05378 | -0,2555 to 0,3531 | 0,7288 | ns | approximate | No |
| CCL4 | 0.08735 | -0,2237 to 0,3823 | 0,5729 | ns | approximate | No |
| CCL5 | -0.1944 | -0,4715 to 0,1177 | 0,2061 | ns | approximate | No |
| TNF-α | 0.1765 | -0,1359 to 0,457 | 0,2518 | ns | approximate | No |
